# First Molecular Evidence and Genetic Characterization of Ovine Herpesvirus 2 in Multiple Animal Species in India

**DOI:** 10.3389/fvets.2021.610178

**Published:** 2021-02-02

**Authors:** Naveen Kumar, Richa Sood, Atul K. Pateriya, E. Venkatesakumar, R. Ramprabhu, Roma Dixit, Sandeep Bhatia, Vijendra Pal Singh

**Affiliations:** ^1^Indian Council of Agricultural Research (ICAR)- National Institute of High Security Animal Diseases, Bhopal, India; ^2^Veterinary College and Research Institute, Tamil Nadu Veterinary and Animal Sciences University (TANUVAS), Namakkal, India; ^3^Veterinary College and Research Institute, Tamil Nadu Veterinary and Animal Sciences University (TANUVAS), Tirunelveli, India

**Keywords:** malignant catarrhal fever, ovine herpesvirus 2, glycoprotein B, India, multiple animals species

## Abstract

Ovine herpesvirus 2 (OvHV-2) is the causative agent of sheep-associated malignant catarrhal fever (SA-MCF), a highly fatal disease syndrome that predominantly affects susceptible hosts of the order *Artiodactyla*. In this study, an in-depth clinico-molecular investigation of SA-MCF disease in a morbid 50-days-old cattle calf (*Bos taurus indicus*) and asymptomatic infection in the in-contact reservoir hosts, sheep (*Ovis aries*), and goat (*Capra hircus*) housed on a farm located in the Southern India is reported. An OIE recommended SA-MCF type-specific PCR confirmed the etiological agent as OvHV-2. The genetic characterization and phylogenetic analyses based on the glycoprotein B (gB) gene indicate that three genetic variants of OvHV-2 had infected the animal cluster of this study. As the OvHV-2 infection eventually lead to the death of the cattle calf, and the fact that its gB sequence carried four unique amino acid substitutions (N169S, L594P, I645V, and V730A), an investigation of these substitutions impact on its stability and molecular flexibility was carried out. The mapping of these amino acid substitutions on the three-dimensional structure of gB coupled with supplementary investigations showed that these substitutions conveyed the molecular flexibility to the gB, at the cost of its stability. Future studies would be to investigate whether these gB substitutions have any impact on membrane fusion activity using a virus-free cell-to-cell membrane fusion assay. The study also highlights the importance of adopting stringent biosecurity measures where mixed animal farming is a common practice.

## Introduction

Malignant catarrhal fever (MCF) is a highly fatal, lymphoproliferative disease syndrome in the susceptible hosts of the order *Artiodactyla*, such as cattle, bison, deer, swine, and water buffalo (1). Currently, six viruses of the genus *Macavirus* of the *Gammaherpesvirinae* subfamily cause the MCF disease and these viruses include alcelaphine herpesvirus-1 (AIHV-1), ovine herpesvirus-2 (OvHV-2), caprine herpesvirus-2 (CpHV-2), alcelaphine herpesvirus-2, ibex malignant catarrhal fever virus, and a virus causing MCF in the deer (2). Of these, OvHV-2 is well-adapted to its natural hosts (sheep and goat) and therefore, causes asymptomatic infection in sheep. In contrast, OvHV-2 is responsible for sheep-associated-MCF (SA-MCF) disease in susceptible hosts such as buffalo (3), cattle (4), deer (5), and rarely in pig (6), and foal (7). The typical clinical signs in the susceptible hosts include pyrexia, anorexia, bilateral corneal opacity, nasal and ocular discharge, ulceration on the mucosa, and neurological manifestations in terminal stages (8). The most common gross pathological changes in the SA-MCF affected animals are erosions of the tracheal and bronchial mucosa, erythema of the turbinate mucosa, congestion and edema of the lungs, and focal white lesions in the kidney. Histopathologically, lymphocytic infiltrations and lymphocytic vasculitis are the usual findings in many tissues and organs. In particular, perivascular cuffing is considered as an important feature for the tentative diagnosis of SA-MCF disease (8).

SA-MCF disease was reported in India in the year 1975 on the basis of histopathological examination (9). The confirmation of SA-MCF disease by OvHV-2 genome detection in sheep and goat (10), captive bison (11), and cattle (12, 13) has since then been reported. The prevalence of OvHV-2 in sheep ranged from 24.44% to 85% in various states in India (10, 12, 14) however, the genetic characterization of OvHV-2 was not reported in any of these studies. In the present study, we report an investigation of SA-MCF disease in a cattle calf and asymptomatic infection in the in-contact reservoir hosts (sheep and goat) that occurred on a farm located at Tirunelveli, Tamil Nadu, India. The genetic characterization of OvHV-2 strains involved in this outbreak was carried out by the sequencing of ORF-8, which encodes glycoprotein B (gB), a key protein essential for virus attachment and entry into the host cells. We found substantial variations in the gB of multiple animal species from India. Furthermore, the supplementary investigations were carried out to assess the impact of amino acid substitutions identified in the cattle calf on the molecular flexibility and stability of gB using the selected computational tools.

## Methods

### Clinical Samples

In October 2017, a morbid 50-days-old cattle calf showing typical SA-MCF disease clinical signs was reported on a farm at the Tirunelveli District, Tamil Nadu, India. The blood samples were collected from the cattle calf and in-contact animals; 4-years-old adult crossbred Jersey cattle (the mother of the cattle calf), Mecheri sheep (*n* = 4, two 6-months-old and other two 5-months-old) and nulliparous 1-year-old non-descript goat (*n* = 1) housed and raised under a semi-intensive system of rearing (partly stall-fed and partly allowed for grazing). The heparinized blood samples were referred to the Indian Council of Agricultural Research-National Institute of High Security Animal Diseases (ICAR-NIHSAD), Bhopal, India for molecular investigations.

### OvHV-2 ORF-8 Gene Sequencing and Phylogenetic Analysis

The genomic DNA was extracted from the peripheral blood mononuclear cells (PBMCs) isolated from the heparinized blood samples using QIAamp DNA Mini Kit (Qiagen) according to the manufacturer's instructions. The presence of OvHV-2 genomic DNA was confirmed by the Office International des Epizooties (OIE) recommended hemi-nested polymerase chain reaction (PCR) as previously described (12, 15). After confirmation, glycoprotein B (gB) which is encoded by ORF-8 of OvHV-2 was amplified using ORF8_F[+] (5′-GGGCCTTTATCTAACGTATGAGA-3′) and ORF8_R[-] (5′-TCACAATGCAAACACTTAYGAGTAA-3′) oligonucleotide sets. The PCR was carried out in a volume of 25 μL containing 12.5 μL of Q5 High-Fidelity 2X Master Mix (New England Biolabs, USA), 10 μM each of oligonucleotide sets, 1.0 μL of template DNA, and remaining nuclease free water. The thermal cycling conditions constituted an initial denaturation at 98°C for 1 min followed by 35 cycles of 30 s at 94°C, 10 s at 50°C, and 3 min at 72°C, and a final extension at 72°C for 10 min. A no-template control was used to confirm that there was absence of DNA contamination in any of the reagents. A specific amplicon of ≈2.8 kb size was detected in 1% agarose gel electrophoresis and was purified by PureLink^TM^ Quick Gel Extraction kit (Life Technologies, USA), cloned in pGEM®-T vector (Promega, USA), and sequenced on an ABI 3730 genetic analyzer (Applied Biosystems, USA). We sequenced only one clone of each PCR product, however, we amplified the full length gB gene by high fidelity polymerase, and each clone was sequenced bi-directionally to reduce the possibility of any error in sequences. The ORF-8 nucleotide sequence data of OvHV-2 detected in two sheep, a cattle calf, and a goat was deposited to the GenBank under accession numbers, MH065713, MH065714, MG972808, and MH065711, respectively.

The nucleotide sequence data of ORF-8 of this study were aligned with the other OvHV-2 ORF-8 sequences retrieved from the National Center for Biotechnology Information (NCBI) database by Clustal W, and the phylogenetic reconstruction was inferred using the Maximum Likelihood statistical method with HKY+G nucleotide substitution model implemented in the MEGA 7 (16). The bootstrap analyses of the trees were achieved with 1,000 replicates of the dataset to determine the robustness of the individual nodes of the tree. The scale bar indicates nucleotide substitutions per site. For each strain, the following data set is furnished: Virus/Species affected/Country/strain name/year of isolation/GenBank accession numbers.

### Assessment of the Impact of Identified Amino Acid Substitutions on gB Conformations and Stability

The four unique amino acid substitutions (N169S, L594P, I645V, and V730A) identified in the OvHV-2 gB detected in the cattle calf were subjected to computational studies to assess the influence of these substitutions on the overall gB stability and flexibility. The 3D models of OvHV-2 gB detected in the sheep and cattle calf were built using I-TASSER (Iterative Threading ASSEmbly Refinement) on templates (PDB: 2gumB, 6escA, and 3fvcA) (17). Using these models, the stability of gB glycoprotein on account of the aforementioned substitutions were assessed and compared using the three distinct protein stability predictors viz. iStable 2.0 which employs machine learning approach builds upon both structure and sequence-based model to predict protein stability (18); DeepDDG which predicts protein stability based on neural networks (19); and DynaMut analyses a protein's stability and dynamics resulting from the vibrational entropy changes (20). If the difference in folding free energy change (ddG) on account of amino acid substitutions gives the results as a positive value (kcal/mol), those substitutions are stabilizing, while the negative values are indicative of de-stabilizing substitutions. Similarly, the positive values of the vibrational entropy energy change (kcal/mol/K) owing to the aforementioned amino acid substitutions are indicative of gain in flexibility, while that of negative values provide rigidity to the protein. The molecular interactions dynamics conveyed by these four amino acid substitutions were visualized by the PyMOL version 2.0.7.

## Results

### The Clinical Picture of the Calf and Treatment Regimen

**Figure 1:**
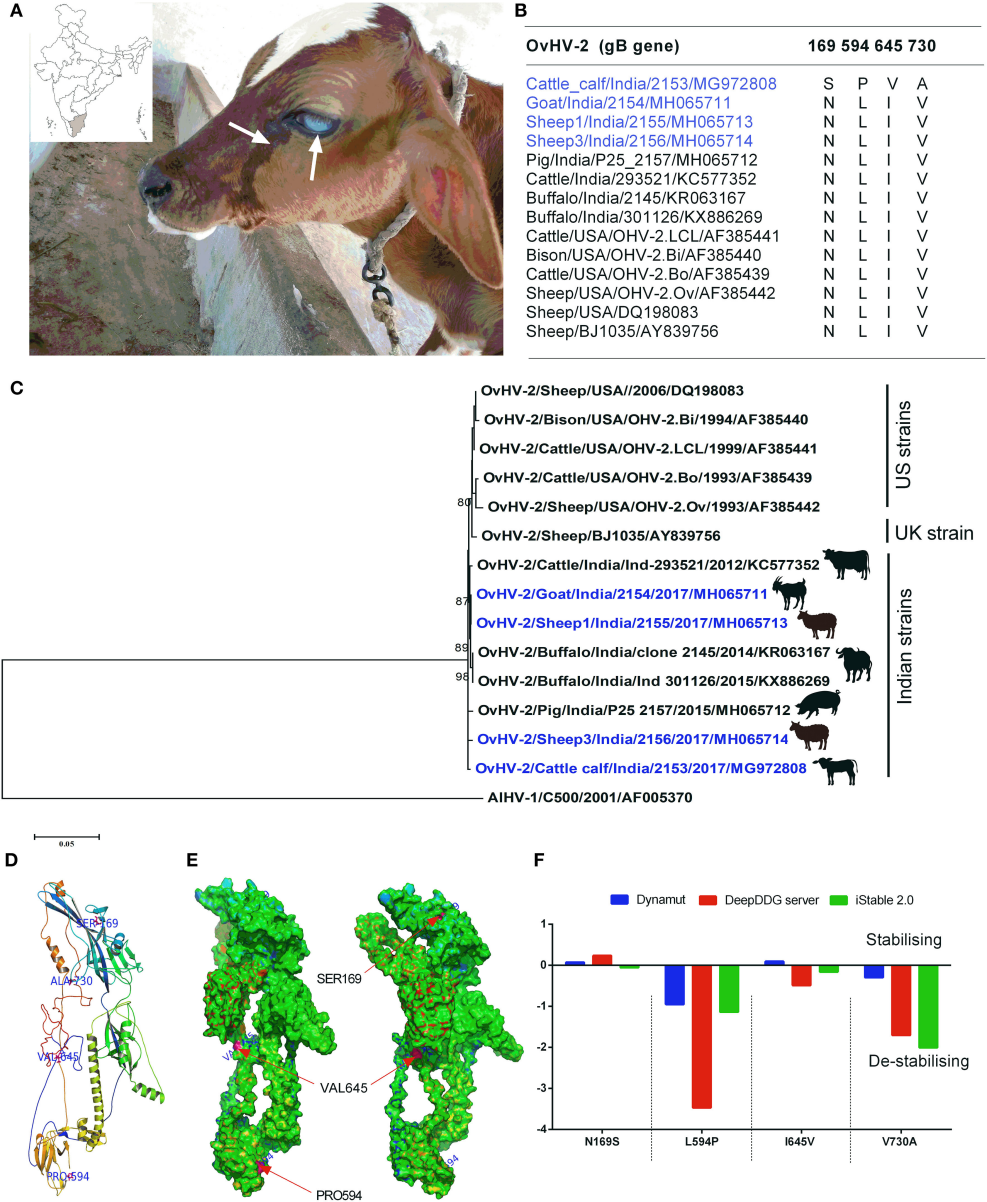
**(A)** A 50-days-old cattle calf showing the typical clinical signs of SA-MCF (bilateral corneal opacity, serous nasal, and ocular discharge), inset shows the geographical location of the study; **(B)** The multiple sequence alignment of OvHV-2 glycoprotein B (gB) involved in the present study (blue colored) along with other OvHV-2 gB sequences retrieved from the NCBI database identified unique amino acid substitutions; **(C)** The maximum likelihood tree of the complete coding ORF-8 (that encodes gB) nucleotide sequences of OvHV-2 strains. The bootstrap values >0.80 are shown. The OvHV-2 strains of the present study are highlighted (blue colored); **(D)** The mapping of four unique amino acid substitutions on the 3D model of calf OvHV-2 gB; **(E)** The classification of amino acid substitutions as solvent-accessible or inaccessible, and mapping them on the 3D model of calf OvHV-2 gB; **(F)** A comparison of differences in folding free energy change (ddG) on account of amino acid substitutions by using three distinct protein stability predictors, iStable 2.0, DeepDDG, and DynaMut servers.

A 50-days-old cattle calf suffering from high fever (40°C), anorexia, serous nasal, and ocular discharge was referred to the Teaching Veterinary Clinical Complex, Veterinary College and Research Institute, Tirunelveli, TamilNadu, India and received a treatment regimen consisting of Ringer's lactate (5 ml/kg i.v.), Dextrose normal saline (5 ml/kg i.v.), Flunixin meglumine (1.1 mg/kg), and a combination of Amoxicillin and Clavulanic acid (12.5 mg/kg BID i.v.) for 7 days. However, the calf did not respond to the treatment regimen and the clinical signs progressed to bilateral epiphora, photophobia, bilateral mucoid nasal discharge, and erosion in the nostrils, muzzle, dental pad, and gums, and the calf eventually succumbed to death on 16 days after the onset of the disease (Figure 1A). In contrast, the sheep, goat, and adult cattle housed in close contact with the diseased calf were asymptomatic. The retrospective investigations revealed that the typical symptoms presented by SA-MCF disease have never been reported in the aforementioned geographical area of Tamil Nadu, India.

### Sequence Diversity and Phylogenetic Analyses of OvHV-2 gB Glycoprotein

The detection of OvHV-2 DNA by OIE prescribed hemi-nested PCR confirmed that the diseased calf suffered from the SA-MCF disease. Of the animals housed with the diseased calf, sheep (*n* = 4) and goat (*n* = 1) were also positive for the OvHV-2 DNA, however, the adult cattle (*n* = 1) was found to be negative for the OvHV-2 DNA. The positive samples from calf, goat, and sheep were subjected to OvHV-2 ORF-8 sequence analysis to compare genetically with the available sequences from the public domain database.

The multiple sequence alignment of the gB identified 24 amino acid substitutions among the five different animal species from India. In the case of OvHV-2 strains of this study, one amino acid substitution (175A) in the gB of sheep1, and goat, four in cattle calf (169S, 594P, 645V, and 730A), and seven in sheep3 (62Y, 76L, 77I, 203N, 610T, 623L, and 633A) were observed. The gB sequence from the SA-MCF-affected calf differs from those of the in-contact animals, suggesting that the calf was infected with a distinct OvHV-2 strain (Figure 1B). The OvHV-2 strains from the SA-MCF susceptible hosts sequenced by us earlier also showed substantial variations in the gB. For example, we noted two amino acid substitutions in buffaloes (398R, and 436R), four in cattle (20T, 101R, 342S, and 696R), and six in pigs (162G, 252A, 292G, 314R, 539G, and 732S) (Supplementary Figure 1). However, it is not clear whether these susbtitutions have any relevance to the susceptibility of the hosts or are the natural variants that appeared due to the virus propagation in reservoir hosts and/or transmission to SA-MCF susceptible hosts.

Furthermore, the gB of OvHV-2 strains of this study showed a high nucleotide sequence identity (99.26–99.81%) with the previously reported Indian OvHV-2 from different animal species, and also to one European (99.03–99.22%), and five US (98.59–99.50%) strains. Most of the Indian OvHV-2 strains formed a discrete cluster. Notably, the gB of OvHV-2 detected in the cattle calf and sheep3 of the this study, and a pig OvHV-2 strain (Pig/India/P25_2157/2015/MH065712)—that had been detected in the Mizoram, a state located in the North-Eastern part of India in the year 2015—formed individual paraphyletic cluster. The remaining two, goat and sheep1 of this study clustered with the buffalo strains, Buffalo/India/301126/2015/KX886269, and Buffalo/India/2145/2014/KR063167, both of which had been reported from the Southern states of India, Andhra Pradesh and Tamil Nadu, respectively in the year 2014–15 (Figure 1C). The gB sequences comparison coupled with the phylogenetic relationships suggests that this cluster of animals housed on a farm were infected by three genetic variants of OvHV-2, with the goat and sheep 1 having identical sequences, while sheep 3 and the SA-MCF-infected calf were distinct from each other and from the goat- sheep1 sequence. The gB sequences of the US established a separate cluster along with the single UK sequence (BJ1035). However, this UK strain (BJ1035) has been shown to be unusual, even within UK MCF cases (21).

### Four Unique Amino Acid Substitutions Identified in Calf OvHV-2 Improves gB Glycoprotein Molecular Flexibility

Since, this is the first time that a cattle calf, within its second month of life, died of OvHV-2 infection, and the fact that its gB sequence is distinct from the in-contact animals, this prompted us to investigate the effect of calf gB substitutions on its stability and molecular flexibility. Structural mapping of amino acid substitutions (N169S, L594P, I645V, and V730A) on the predicted three-dimensional structure of calf OvHV-2 gB showed that three substitutions (N169S, L594P, and I645V) are located on the random coil, while the fourth one (V730A) on the helix (Figure 1D). Of these, two substitutions (L594P, and I645V) are solvent accessible, while the other two substitutions (N169S, and V730A) are solvent inaccessible (Figure 1E). Of particular, the solvent accessible (L594P), and inaccessible (V730A) substitutions in the gB are de-stabilizing, a consistent result obtained on comparing the three distinct protein stability predictors (Figure 1F).

**Figure 2:**
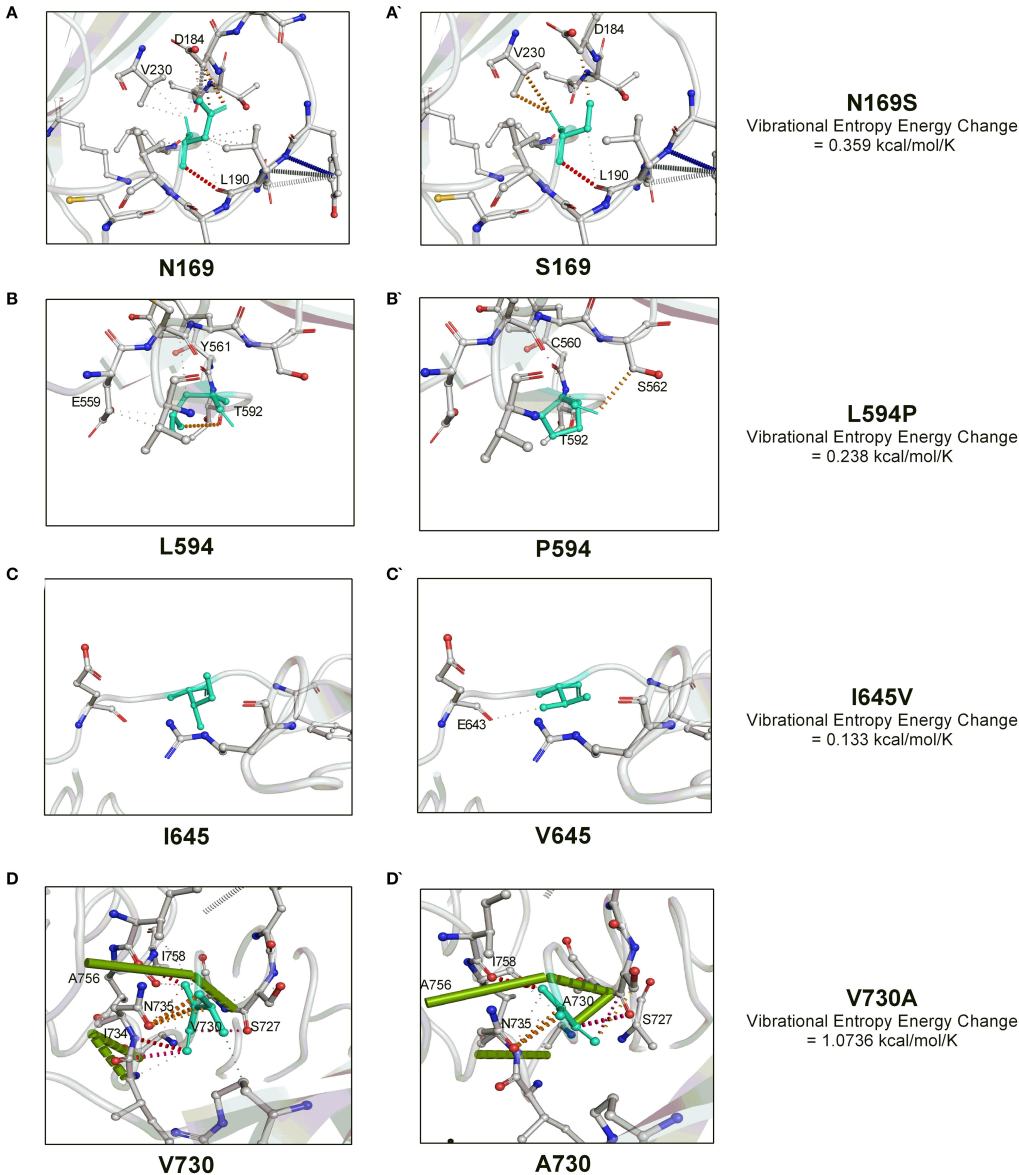
The molecular interactions dynamics conveyed by four unique amino acid substitutions (N169S, L594P, I645V, and V730A) within the OvHV-2 gB. The vibrational entropy change on account of a particular amino acid substitution is also shown. The molecular interactions dynamics were visualized by the PyMOL.

We next investigated the influence of these substitutions on the gB flexibility and thereon molecular interactions with the neighboring amino acids. A positive value of vibrational entropy energy change, indicative of an increase in molecular flexibility on account of substitution, was noted for all the four substitutions, the highest being for the V730A substitution (Figure 2). The key molecular interactions variations as a result of the substitution of a particular amino acid in the gB are presented in Figure 2. In this, N169S substitution allows the institution of a weak H-bond with V230 by replacing with D184 (Figures 2A,A'). L594P substitution leads to the loss of hydrophobic interactions with Y561, and a weak H-bond with E559, with the establishment of carbonyl interactions with C560 and a weak H- bond with S562 (Figures 2B,B'). I645V substitution establishes a weak H-bond with E643 (Figures 2C,C'). V730A substitution leads to loss of carbonyl interactions with I734, two amide bonds between I734-N735 and I734-G732, and the establishment of an amide bond between G732-N735 (Figures 2D,D'). Overall, the variations observed in the molecular interactions due to the aforementioned substitutions conveyed the molecular flexibility to the gB glycoprotein.

## Discussion

In this study, we have described a clinico-molecular investigation of SA-MCF disease in a cattle calf on a farm located in Tirunelveli, TamilNadu, India. The OvHV-2 DNA in a cattle calf, four sheep and one goat was confirmed by the OIE prescribed hemi-nested PCR. Of the OvHV-2 DNA positive animals, the sheep and goat were asymptomatic, while the cattle calf showed the typical clinical signs of SA-MCF (high fever, bilateral corneal opacity, and mucoid ocular and nasal discharge) and died after 16 days of onset of the disease. However, the mother of the calf, a Jersey cattle kept in close contact with the diseased calf on the same farm was negative for OvHV-2 DNA. Previous studies from India, although limited, reported the presence of OvHV-2 in sheep (10), captive bison (11), and cattle (12, 13), however, most of these studies did not include genetic characterization of OvHV-2. Therefore, the present study is the first to report molecular detection and genetic characterization of OvHV-2 in multiple animal species (sheep, goat, and cattle calf) in India. In addition, this study offers the genetic analysis of largest collection of full length OvHV-2 gB sequences globally, including six sequences from the US, and one from UK.

The sequencing and phylogenetic analyses of OvHV-2 gB glycoproteins showed that all Indian OvHV-2 strains, including the strains reported in the current study, grouped together apart from the US and UK sequences, suggesting that the evolution of OvHV-2 in reservoir hosts and subsequently dead-end transmission to SA-MCF susceptible hosts in India. However, the separate clustering of OvHV-2 strains reported in this study—individual paraphyletic cluster by cattle calf, sheep3 and a pig OvHV-2 strain, and monophyletic clustering of goat and sheep1 of this study with the buffalo OvHV-2 strains—with the strains detected in the distantly separated geographical locations along with distinct amino acid substitutions (one each in goat and sheep1, four in calf, and seven in sheep 3) indicates that three genetic variants of OvHV-2 had infected the animal cluster of this study (Figure 1C, Supplementary Figure 1).The retrospective investigations revealed that four sheep were introduced in the farm recently and were purchased from a weekly livestock market (where different animals from the nearby villages and districts are brought together). The sequence analysis has already showed that the two sheep (sheep 1 and sheep 3) had distinct OvHV-2 strains, while the gB of remaining two sheep could not be sequenced due to low volume of the samples. It is possible that the calf acquired the OvHV-2 infection from either of these two sheep, because calf had no history of common grazing with animals of other farms. Based on a few molecular epidemiology studies (by RT-PCR) on OvHV-2 that have been conducted so far in India, the prevalence of OvHV-2 in sheep varied from 24.44% to 85%, the highest being in the Northern part of India and the lowest in the Southern part of India (10, 12, 14). It may be noted that these differences arising from the different grographical regions may likely be due to false-negative results in RT-PCR assays, due to the low viral load in blood of the latently infected individual animals from reservoir species (sheep and goats). Therefore, employing a reliable serological assay for the detection of OvHV-2 infection of sheep and goats would allow a more accurate estimation of prevalence. Frequent migrations of animals across the state borders coupled with lack of awareness and asymptomatic carrier status in sheep permits the easy spread of SA-MCF disease. The housing of OvHV-2 reservoir/asymptomatic carrier host (sheep and goat) with the susceptible host species (buffalo and cattle) is a regular practice in India, which furthermore allows frequent cross-species transmission among the reservoir hosts, and dead-end transmission to SA-MCF susceptible hosts.

A typical gammaherpesvirus gains entry into the host cells *via* binding of one or more viral glycoproteins to the cellular receptor(s), followed by the fusion of viral envelope with the host cell membrane. Glycoprotein B (gB) plays an important role in the entry of a gammaherpesvirus into the host cells *via* heparan sulfate and/or α3β1 integrin (22, 23). Together, gB, glycoprotein L (gL), and glycoprotein H (gH) of OvHV-2 are necessary for membrane fusion and are considered as the core fusion machinery (24). Given the essentiality of gB glycoprotein of OvHV-2 in entry to the host cells, we sequenced OvHV-2 gB detected in cattle calf, sheep, and goat and compared with the available gB sequences in the public domain database. Twenty four amino acid substitutions in the eight gB encoding sequences were noted among the five different species from India. Indeed, all these sequence variations were obtained from the natural OvHV-2 infection. These results suggests that these variations must be part of the functional gB proteins in the reservoir/SA-MCF susceptible hosts with structural consequences (on account of these substitutions) that are either tolerated or complemented in conjugation with other glycoproteins. As a matter of fact, despite a much lower degree of sequence identity, the OvHV-2 gB gene has been shown to complement a deletion of the gB homolog in a chimeric AlHV-1 (25). This suggests that a wide range of amino acid substitutions in non-essential residues within the gB may be tolerated without compromising the function.

As the OvHV-2 infection eventually lead to the death of a cattle calf, and the fact that it's gB sequence is distinct from the in-contact animals (suggesting a different source of infection), this prompted us to investigate the effect of calf gB substitutions (N169S, L594P, I645V, and V730A) on its stability and molecular flexibility. Although significant progress has been made in the detection and understanding of SA-MCF disease, the lack of a permissive cell culture system for OvHV-2 limits the studies of viral entry mechanism and cell tropism. Using the selected computational tools, we showed that these amino acid substitutions provide the molecular flexibility to the gB glycoprotein, at the expense of its stability. Future studies would be to investigate whether these gB substitutions have any impact on membrane fusion activity using a virus-free cell-to-cell membrane fusion assay.

There is no effective treatment or vaccine for SA-MCF to date. The prognosis of the disease in susceptible animals is typically serious. Hence, the promising practical approach to contain the transmission of OvHV-2 is to avoid the direct contact of the reservoir hosts (sheep and goat) to the susceptible hosts. Since the mixed livestock farming is a common practice in India, separate housing and feeding combined with distinct grazing schedule for different animal species would be a realistic and economical strategy to break the cross-species transmission of OvHV-2.

## Data Availability Statement

The datasets presented in this study can be found in online repositories. The names of the repository/repositories and accession number(s) can be found in the article/Supplementary Material.

## Author Contributions

EV and RR provided the samples. NK, RS, AP, and RD performed experiments. NK and RS performed the computational analysis and analyzed the data. NK, RS, SB, and VS wrote the manuscript. All authors contributed to the article and approved the submitted version.

## Conflict of Interest

The authors declare that the research was conducted in the absence of any commercial or financial relationships that could be construed as a potential conflict of interest.

## Supplementary Material

Supplementary Figure 1 The multiple sequence alignment of the complete gB of OvHV-2 strains of the present study along with other OvHV-2 strains retrieved from the NCBI database.
